# Cancer cell redirection biomarker discovery using a mutual information approach

**DOI:** 10.1371/journal.pone.0179265

**Published:** 2017-06-08

**Authors:** Kimberly Roche, F. Alex Feltus, Jang Pyo Park, Marie-May Coissieux, Chenyan Chang, Vera B. S. Chan, Mohamed Bentires-Alj, Brian W. Booth

**Affiliations:** 1 Department of Genetics and Biochemistry, Clemson University, Clemson, South Carolina, United States of America; 2 Institute for Biological Interfaces of Engineering, Clemson University, Clemson, South Carolina, United States of America; 3 Department of Biomedicine, University of Basel, University Hospital Basel, Basel, Switzerland; 4 Friedrich Miescher Institute for Biomedical Research, Basel, Switzerland; 5 Department of Biological Sciences, Clemson University, Clemson, South Carolina, United States of America; 6 Department of Bioengineering, Clemson University, Clemson, South Carolina, United States of America; "Sanford-Burnham Medical Research Institute", UNITED STATES

## Abstract

Introducing tumor-derived cells into normal mammary stem cell niches at a sufficiently high ratio of normal to tumorous cells causes those tumor cells to undergo a change to normal mammary phenotype and yield normal mammary progeny. This phenomenon has been termed cancer cell redirection. We have developed an *in vitro* model that mimics *in vivo* redirection of cancer cells by the normal mammary microenvironment. Using the RNA profiling data from this cellular model, we examined high-level characteristics of the normal, redirected, and tumor transcriptomes and found the global expression profiles clearly distinguish the three expression states. To identify potential redirection biomarkers that cause the redirected state to shift toward the normal expression pattern, we used mutual information relationships between normal, redirected, and tumor cell groups. Mutual information relationship analysis reduced a dataset of over 35,000 gene expression measurements spread over 13,000 curated gene sets to a set of 20 significant molecular signatures totaling 906 unique loci. Several of these molecular signatures are hallmark drivers of the tumor state. Using differential expression as a guide, we further refined the gene set to 120 core redirection biomarker genes. The expression levels of these core biomarkers are sufficient to make the normal and redirected gene expression states indistinguishable from each other but radically different from the tumor state.

## Introduction

Cancer initiation and progression is a complex genetic disease where mutations and epigenetic alterations in cells lead to inactivation of tumor suppression genes, activation of oncogenes, and impact DNA repair processes [1–2]. The accumulation of genetic mutations often leads to deregulation of proliferative signaling pathways and suppression of pro-differentiation apoptotic pathways. The differential expression of signaling pathways affects not only the mutated cells but also impacts surrounding cells through intercellular communication involving biophysical and biochemical modes of signaling. Intercellular communication is not a one-direction process, as surrounding cells are known to influence tumor cell activity [3].

Normal mammary microenvironments, or somatic stem cell niches, have been shown to direct the cell fates of non-mammary stem cells [4–7]. When non-mammary stem cells are incorporated into reforming mammary stem cell niches during mammary tissue regeneration the non-mammary stem cells adopt a mammary phenotype and provide progeny to a functional reconstituted mammary gland *in vivo*. When tumor-derived cells are incorporated into reforming normal mammary stem cell niches the tumor cells also adopt a normal mammary phenotype and provide differentiated cell progeny to the normal mammary outgrowth [8–10]. This phenomenon has been termed cancer cell redirection. Cancer cell redirection is determined on the ratio of cancer cells to normal mammary epithelial cells present. Successful redirection of human breast cancer cells and mouse mammary tumor cells occurs at a ratio of 1 cancer cell for every 50 normal epithelial cells while redirection of embryonal testicular carcinoma cells can occur at a ratio of 1 cancer cell for every 10 normal cells. When mouse mammary-tumor derived cells that overexpress erbB2 are redirected, the auto-phosphorylation of the erbB2 tyrosine kinase is attenuated while total expression of erbB2 is not impacted [11]. Reduced erbB2 phosphorylation is one mechanism involved in cancer cell redirection and detection of reduced receptor activity serves as a biomarker of cancer cell redirection.

The genetic profiles of insipient cancer cells change from normal expression patterns to ultimately their cancer cell profile [12]. Breast cancers have been divided into six main groups based on genetic profiles with additional subgroups being added upon discovery of new genetic profiles. The six main groups are 1) Luminal A, 2) Luminal B, 3) HER2 enriched, and 4) claudin low, 5) normal-like, and 6) basal-like [13]. Amplification and overexpression of HER2 is observed in 20–30% of human breast cancers and is inversely correlated with patient survival [14–16]. Within these complex differential gene expression patterns lie the causal genetic mechanisms underlying tumorigenesis and possibly cancer cell redirection.

We have developed an *in vitro* system of cancer cell redirection that employs the 1:50 ratio of erbB2-overexpressing cancer cells to normal cells [11]. This *in vitro* system has been validated as an alternative to the *in vivo* model. Using our *in vitro* system of cancer cell redirection, we investigated the genetic profiles of erbB2-overexpressing mammary tumor-derived cells as they undergo the redirection phenomenon. In this report, we present transcriptome profiling results from multiple mouse sorted cell groups that model normal, redirected, and tumor states. Then we describe the application of a mutual information approach to identify relevant molecular signatures (biomarker packages) that appear to be involved in cancer cell redirection.

## Materials and methods

### Cell culture

Establishment of the MMTV-neu cell lines is described elsewhere [11]. MMTV-neu cells were maintained in DMEM supplemented with 10% FBS and 1% antibiotic/antimycotic. COMMA-D cells were grown in DMEM/F12 with 2% FBS, 0.1M HEPES, 1% antibiotic/antimycotic, 10 **μg** /ml insulin, 5 ng/ml EGF and 4 **μg**/ml gentamicin added. Co-cultures of MMTV-neu and COMMA-D cells were grown in COMMA-D medium. All cultures were grown at 37°C and 5% CO_2_.

### Magnetic sorting

**Fig 1:**
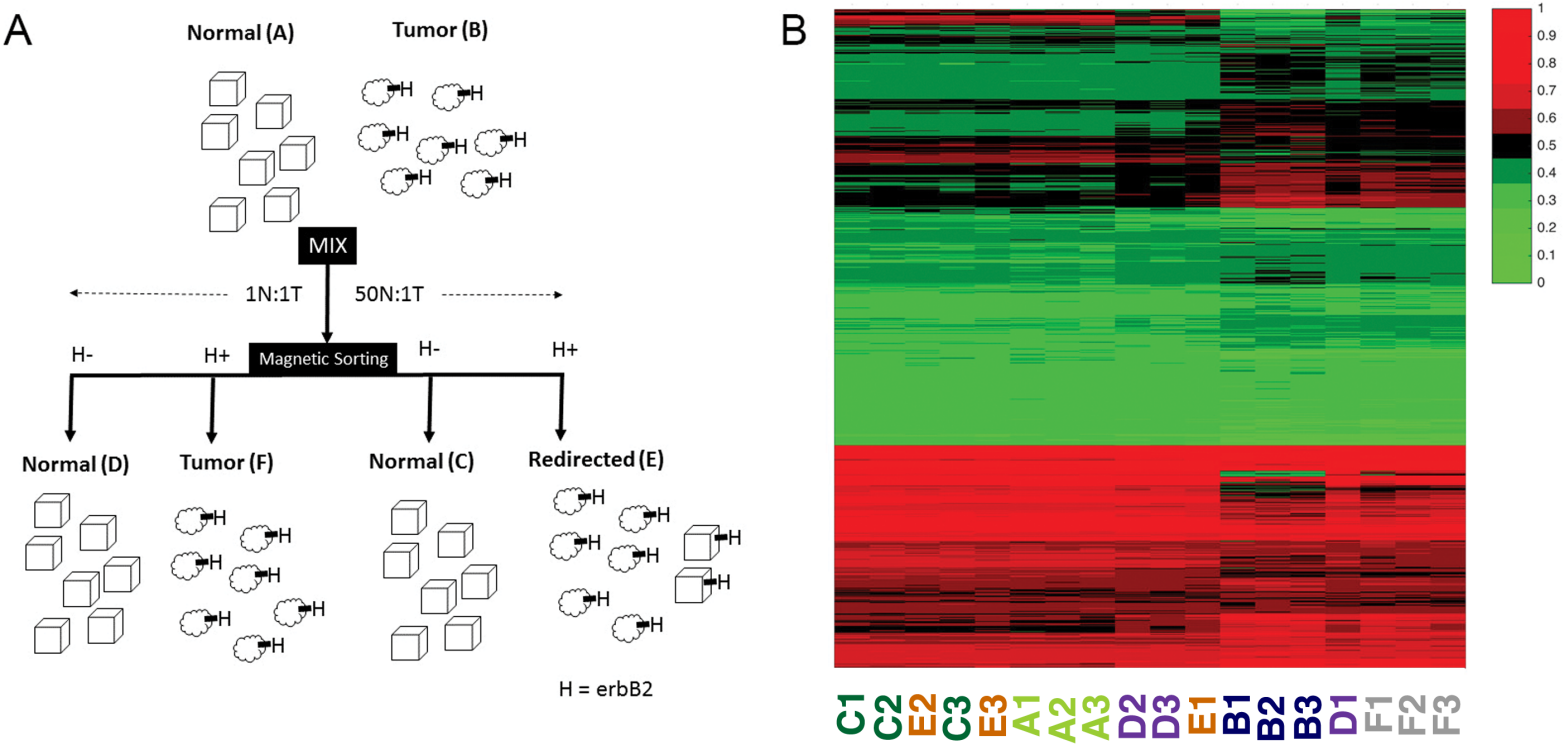
The experimental system and global gene expression pattern. **A**: The experimental system consists of six cell groups; normal (A) and tumor (B) cells were mixed in either a 1:1 or 50:1 (normal:tumor) ratio and cultured for 4 days, then magnetically sorted based on expression of erbB2 into four resultant groups: normal (D) or tumor (F) from the 1:1 mixing and normal (C) and redirected tumor (E) from the 50:1 mixing; **B**: Heatmap of all transcripts present in the microarray, log_2_ expression values normalized by column; columns are identified at the bottom of the figure with their cell group classification and replicate number.

The protocol used for magnetic sorting is described elsewhere [8]. After 4 days in culture cells were detached by trypsin, washed once with PBS and resuspended in 100 **μ**l of diluent containing primary antibody (1:50; anti-erbB2, Miltenyi) and incubated for 30 min at 4°C. The cells were then washed 2 x 10 min and resuspended in 100 **μ**l of diluent containing biotinylated secondary antibody (1:100) and incubated for 15 min at 4°C. The cells were then washed 2 x 10 min and resuspended in 80:20 **μ**l mix of diluent and anti-biotin magnetic beads (Miltenyi) and incubated for 15 min at 4°C. The cells were then washed 2 x 10 min and resuspended in 500 **μ**l of running buffer and separated through a LS column (Miltenyi). The erbB2-positive and erbB2-depleted fractions were collected (Fig 1A).

### RNA isolation

Total RNA was isolated from the sorted samples using an RNAaqueous Micro kit (Ambion, Austin, TX) that utilizes glass fiber filter column purification. Isolated total RNA was then treated with DNase (Qiagen, Valencia, CA) to remove any DNA contamination. The concentration of RNA was determined using a NanoDrop and the RNA integrity was analyzed with a RNA nano chip on a Bioanalyzer (Agilent Technologies, Santa Clara, CA). Total RNA was isolated from three independent experiments performed sequentially.

### Microarray analysis

A total of eighteen samples (6 groups in triplicate) were run on Mouse gene 1.0 ST Arrays from Affymetrix (http://www.affymetrix.com). Samples were RMA normalized and log2 gene expression matrices processed using R v3.1.2 and the following Bioconductor v3.1 packages [17]: *affy* [18], *limma* [19], and oligo [20] according to standard procedures [21]. The experimental design for differential gene expression with *limma* was CN = c(0,0,0,1,1,1), TR = c(1,1,1,0,0,0). Differential gene expression results between all combinations of cell groups are available in S1 Table. The microarray CEL files were deposited in the NCBI GEO database under accession # GSE89963.

### qRT-PCR validation

To validate microarray results we selected three genes: *AREG*, *ERBB2*, and *THBS1* for qRT-PCR analysis. The housekeeping gene *GAPDH* was used as a reference gene to normalize the expression of each test gene. Primers used for each gene are listed in S2 Table. RNA was reverse-transcribed to cDNA using the Ambion Retroscript kit two-step protocol. The quantity and quality of the RNA was determined using a Nanodrop 2000. The cDNA was analyzed using the QuantiTech SYBR Green RT-PCR Kit (Qiagen, Valencia, CA) on the Step One Plus (Applied Biosystems, Grand Island, NY). Relative expression was determined using the **ΔΔ**Ct method with GAPDH as the reference gene. Results of the validation are presented in S1 Fig.

### Data analysis

Data was collected as RNA sequence microarray measurements from three repeated experiments involving six cell groups, A—F (Fig 1A). The RNA normalized gene expression matrix (GEM; available accession # GSE89963) consisted of intensity measurements of 35,556 probe sets for the cell groups, three replicates each of A through F, yielding 18 columns. The range of the data was compressed by log_2_ transformation, yielding an expression range from 3.582 to 14.052. All calculations were performed in *MATLAB*. Mutual information (MI), using units of “bits,” was used to describe the similarity of the cell groups to each other and of subsets of genes to each other. Here, the term “information” (or alternatively, “entropy”) reflects its usage in information theory, as a measure of uncertainty about a distribution [21]. *Mutual information* is a method of measuring the symmetric dependence of two variables. It is not dissimilar to Pearson's correlation, in this sense. Unlike Pearson's correlation however, mutual information, defined logarithmically, is not limited in its sensitivity to the measurement of linear relationships between groups. [21]. We utilized an existing MI toolbox for *MATLAB* [22].

### Visualization

Cell groups were visualized using classic multidimensional scaling (CMDS) and the *cmdscale* function in *MATLAB*. For this, a dissimilarity matrix (DM) was built based on inverse MI. MI was calculated for each combination of cell group-replicates, column pairs from the gene expression matrix (GEM), and maximum entropy for the set size was divided by this MI value to give a dissimilarity score. CMDS was used to build an approximate picture in 1-dimensional space of the “spread” of the expression profiles for each cell group and later, to visualize the relationship, in 3-dimensional space, of the similarity of the cell groups to each other based on subsets of genes.

### Confocal imaging

Cultures were grown on chamber slides and fixed with 4% paraformaldehyde for 10 min at room temperature, rinsed with PBS then stained using standard protocols. Briefly, the fixed cells were permeabilized with 0.1% Triton X-100 then blocked using 10% serum. The primary antibodies used were anti-Mapk1 (1:800), anti-phospho-Neu (1:50)(Santa Cruz Biotech), anti-Notch2 (1:200)(Cell Signaling Technologies); each was applied and incubated overnight at 4°C. Secondary antibodies were conjugated to Alexa 488 or Alexa 568 (1:200)(Life Technologies) and incubated for 60 min at room temperature. Slides were coverslipped with ProLong Antidfade mounting media containing DAPI (Life Technologies). Images were collected using a Leica TCS SPE confocal microscope at the same laser and acquisition setting. To ensure the laser power and imaging settings were not acquiring autofluoresence, a comparison image was generated from using an unstained sample. Images were analyzed using Image J and Adobe Photoshop.

### Western blotting

Protein lysates were collected using M-PER mammalian protein extraction reagent with protease and phosphatase inhibitors added (Thermo Scientific; Rockford, IL). Lysates were cleared at 10,000 rpm for 10 min at 4°C then transferred to new tubes and frozen at -20°C until analysis. Protein concentration was determined using a BCA assay. Samples were combined with sample running buffer and boiled for 10 min. Proteins were separated on a 4–20% gel, transferred to nitrocellulose then probed using anti-**β**-actin and anti-Notch2 followed by HRP-conjugated secondary antibodies (all Cell Signaling Tech). Target proteins were detected using the chemiluminescent reagent Lumiglo (Cell Signaling) and visualized using FluorChemM (Cell Biosciences; Wallingford, CT).

## Results

### Sample collection

Mammary tumor-derived cells that overexpress erbB2 were co-cultured with normal mammary epithelial cells in our in vitro model of cancer cell redirection. The different ratio cultures were magnetically sorted into erbB2^+^ and erbB2^-^ fractions (Fig 1A). Each fraction was assigned a Group designation (A-F) (Fig 1A). RNA collected from each fraction was then arrayed. Generated heatmaps of all transcripts are presented in Fig 1B.

### MI-based molecular signature discovery

**Fig 2:**
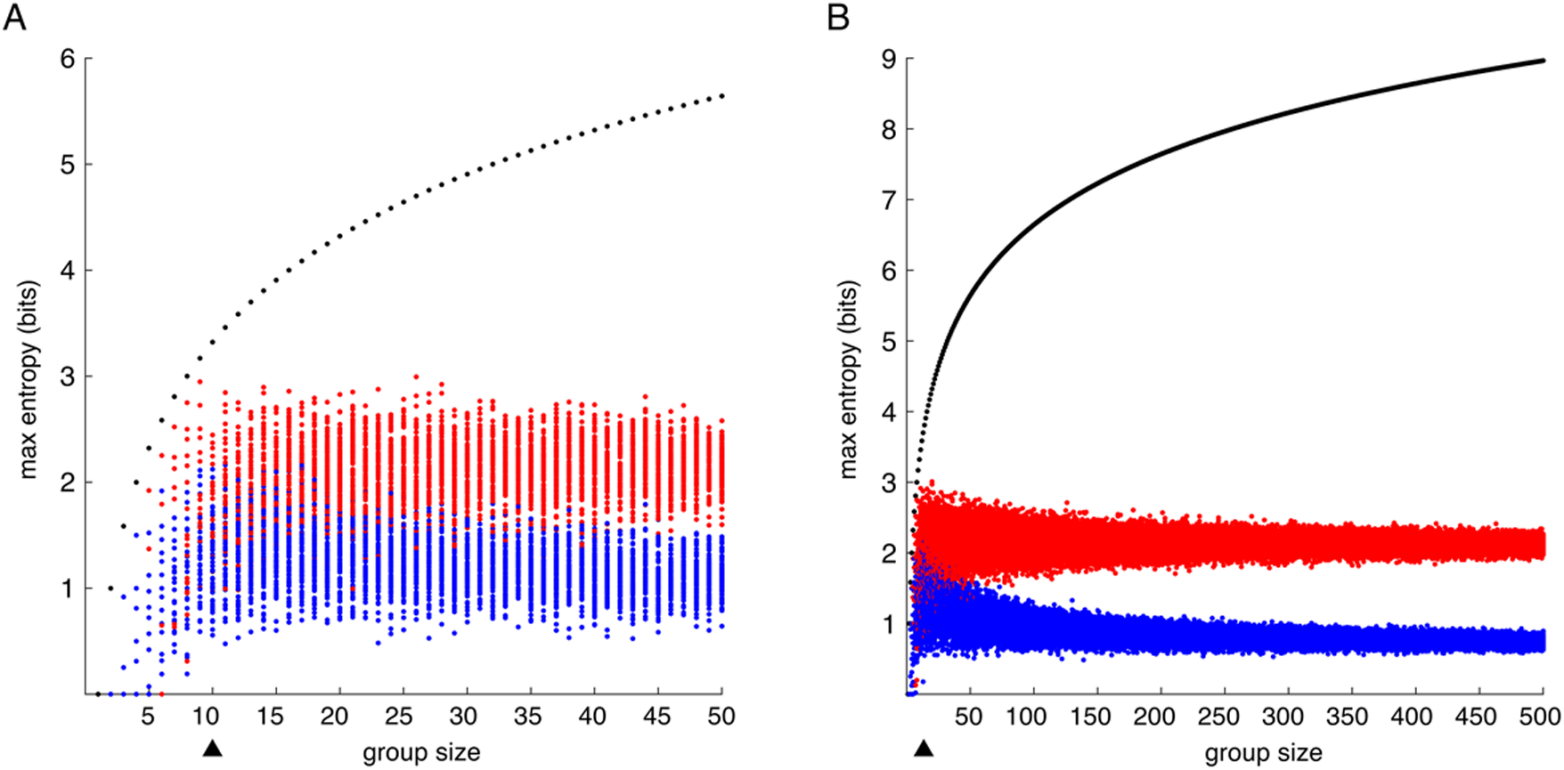
Mutual information performance and set size. MI and entropy for molecular signatures of increasing size. Points in black represent maximum entropy for a given set size–maximum possible information; points in red are MI values of random samples sets from columns A_1_ and A_2_ of the GEM (likely to be more similar as they belong to the same cell group); blue measurements are from columns A_1_ and B_1_ of the GEM (likely to be less similar); Triangle markers represents the molecular signature size minimum used in our calculations; molecular signatures below this size were excluded from consideration. **A**: zoomed group size 1 to 50; **B**: a larger plot of group size 1 to 500.

In the interest of exploring the behavior of genes with shared action, 13,311 gene lists, or molecular signatures, from the Broad Institute Molecular Signatures Database were evaluated. To transform the molecular signatures to sets of values from the gene expression matrix (GEM), each signature was translated first into official gene identifiers that were translated into probe set identifiers in the GEM. Not all genes in all molecular signatures were represented in our GEM. This gave each molecular signature represented by an *N* x 18 expression matrix, a subset of the GEM. Molecular signatures with gene membership *N* of 10 or fewer were excluded as being too small to demonstrate reliable mutual information (MI) measurements (Fig 2). Only sets of a size equal to or greater than the range of the data were included for consideration. We attempted to extend each molecular signature by inputting the sets into genemania.org and filtering linked genes by genetic, physical, and co-expression gene interactions. The extended molecular signatures sets are available in S3 Table. Molecular signatures that maximized the ratio of MI between normal and redirected cell groups (groups A and E) to MI between redirected and tumor cell groups (E and B) were determined by this equation:

$$\frac{MI(A,E)}{MI(E,B)}$$

This ratio was calculated for each molecular signature to build a distribution of such values. A significance threshold was defined using the Bonferroni correction [23]. The molecular signatures were filtered by minimum size and those with MI(A,E)/MI(E,B) of zero were excluded. Of the 13,311 molecular signatures, 12,527 remained after this filtering. Bonferroni correction yielded a significance threshold of 7.983 x 10^−7^. This corresponded to a lowest significant MI ratio value of 5.817. Molecular signatures whose MI ratio values were above or equal to this threshold were selected as pathways statistically significant with respect to tumor cell redirection.

### Visualization

**Fig 3:**
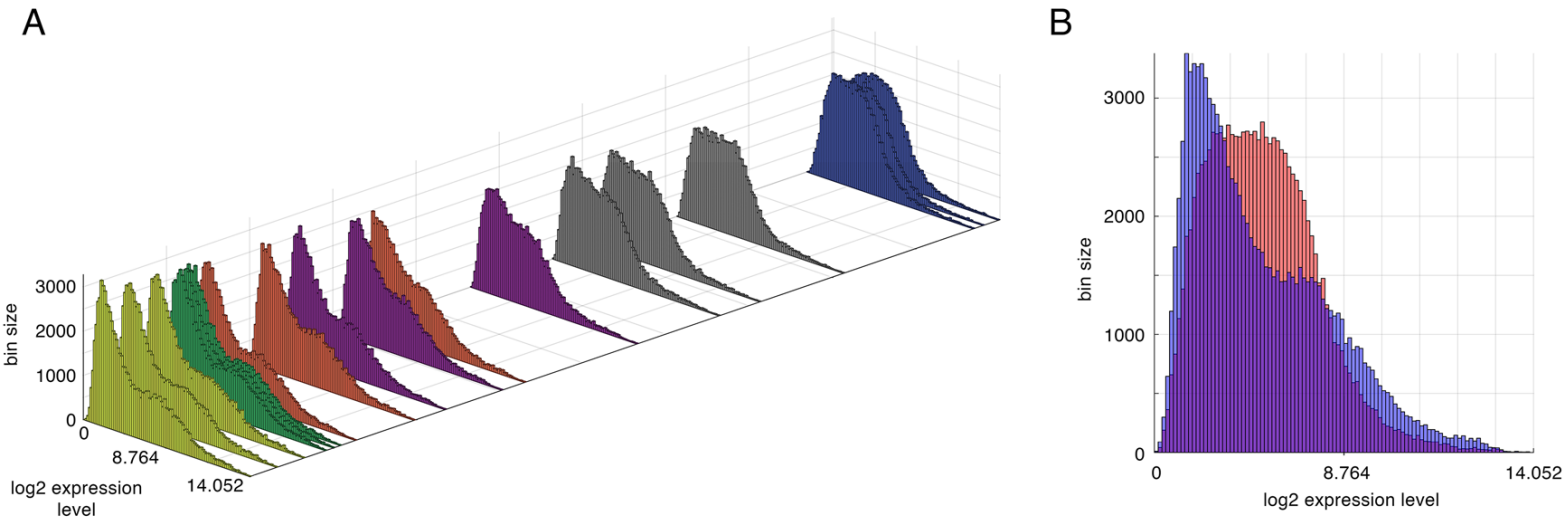
Distribution of expression levels for each cell group. **A**: Expression level distributions ordered in one dimension on the basis of dissimilarity by mutual information. Yellow = normal (cell group A), orange = redirected (E), blue = tumor (B). Order of cell group-replicates, from bottom-left to top-right: A_3_, A_1_, A_2_, C_3_, C_2_, C_1_, E_2_, E_3_, D_3_, D_2_, E_1_, D_1_, F_3_, F_2_, F_1_, B_1_, B_2_, B_3_. **B**: Expression level distributions for all cell group E replicates (blue) superimposed on distributions for all B replicates (red).

Distributions of expression levels for each cell group showed a remarkable pattern (Fig 3A). Tumor cell groups (B, F) generally exhibited a single peak while a weakly bimodal shape characterized the distributions of normal (A, C, D) and redirected (E) cell groups (Fig 3B). Using CMDS, we rendered the distributions in the single dimension of greatest dissimilarity as calculated by mutual information (Fig 3A). Note the progressive change in distribution shape along this axis.

### MI-based molecular signature discovery

Mutual information ratios were of interest because information content increases with set size. By measuring relative relationships in the dataset, we normalized for the effect of set size on the measurements. We were most interested in the Broad molecular signatures that maximized MI(A,E)/MI(E,B)–i.e. those that demonstrated high mutual information between cell groups A and E and low MI between E and B, interpreting these to be pathways that showed similar patterns of expression in A and E (normal and redirected) and dissimilar patterns of expression in E and B (redirected and tumor).

Based on this ratio, molecular signatures above the threshold of significance were identified. Of the molecular signatures evaluated, 20 exceeded this threshold on the upper limit of the distribution. Those molecular signatures, which demonstrate the greatest whole-group transformations in expression of redirected cells along the tumor-to-normal spectrum, are listed in Table 1. MI ratio results for all molecular signatures evaluated are available in S4 Table.

**Table 1:** Molecular signatures that demonstrate the greatest MI ratio.

Molecular Signatures	MI Ratio	MSDB Description
REACTOME_SOS_MEDIATED_SIGNALING	12.726	Genes involved in SOS-mediated signaling
BIOCARTA_CERAMIDE_PATHWAY	9.050	Ceramide signaling pathway
REACTOME_NOTCH_HLH_TRANSCRIPTION PATHWAY	8.565	Genes involved in Notch-HLH transcription pathway
MOOTH_FFA_OXYDATION	7.557	Genes involved in free fatty acid oxidation
REACTOME_PROCESSIVE_SYNTHESIS_ON_THE_LAGGING_STRAND	7.217	Genes involved in Processive synthesis on lagging strand
LANDIS_BREAST_CANCER_PROGRESSION_LP	7.141	Genes upregulated in preneoplastic mammary tissues
GOTZMANN_EPITHELIAL_MESENCHYMAL_TRANSITION_UP	7.134	Genes upregulated during EMT
INGA_TP53_TARGETS	6.573	Genes with promotors containing TP53 response elements
NIKOLSKY_BREAST_CANCER_21Q22_AMPLICON	6.494	Genes within amplicon 21q22
REACTOME_POL_SWITCHING	6.490	Genes involved in polymerase switching
CROMER_METASTASIS_DN	6.381	Metastatic markers of head and neck cancers
SA_PROGRAMMED_CELL_DEATH	6.331	Genes involved in apoptosis
BOGNI_TREATMENT_RELATED_MYELOID_LEUKEMIA_DN	6.153	Genes down-regulated in ALI
KEGG_SNARE_INTERACTIONS_IN_VESICULAR_TRANSPORT	6.134	Genes involved in SNARE interactions
FLECHNER_BIOPSY_KIDNEY_TRANSPLANT_OK_VS_DONOR_DN	5.964	Genes down-regulared in normal kidneys
MOSERLE_IFNA_RESPONSE	5.842	Upregulated genes in ovarian progenitor cells in response to IFN-α
HUANG_DASATINIB_RESISTANCE_UP	5.836	Genes with expression that correlates with dasatinib treatment
KEGG_BASE_EXCISION_REPAIR	5.835	Genes involved in base excision repair
PID_CERAMIDE_PATHWAY	5.824	Genes in the ceramide signaling pathway
MILI_PSEUDOPODIA_CHEMOTAXIS_DN	5.817	Genes down-regulated in response to LPA

Each of the significant molecular signatures was extended with additional interacting genes, associated with those already present in the molecular signature. The additional genes were added on the basis of significant genetic, co-expression, or physical interaction found in the genemania.org database and significant differential gene expression between cell groups A (normal) and E (redirected) from B (tumor). When MI ratios were calculated for these molecular signatures extended with differentially expressed (p<0.0001) interacting genes, the effect was an improvement in MI(A,E)/MI(E,B) in a majority of cases where new genes were incorporated (REACTOME_SOS_MEDIATED_SIGNALLING, GOTZMANN_EPITHELIAL_TO_MESENCHYMAL_TRANSITION_UP, INGA_TP53_TARGETS, CROMER_METASTASIS_DN, and MILI_PSEUDOPODIA_CHEMOTAXIS_DN). In the case of the most improved molecular signature, REACTOME_SOS_MEDIATED_SIGNALLING, the MI ratio was increased from 12.726 to 15.812. Results of this extension for all molecular signatures, at a variety of stringency levels, are available in S5 Table.

**Fig 4:**
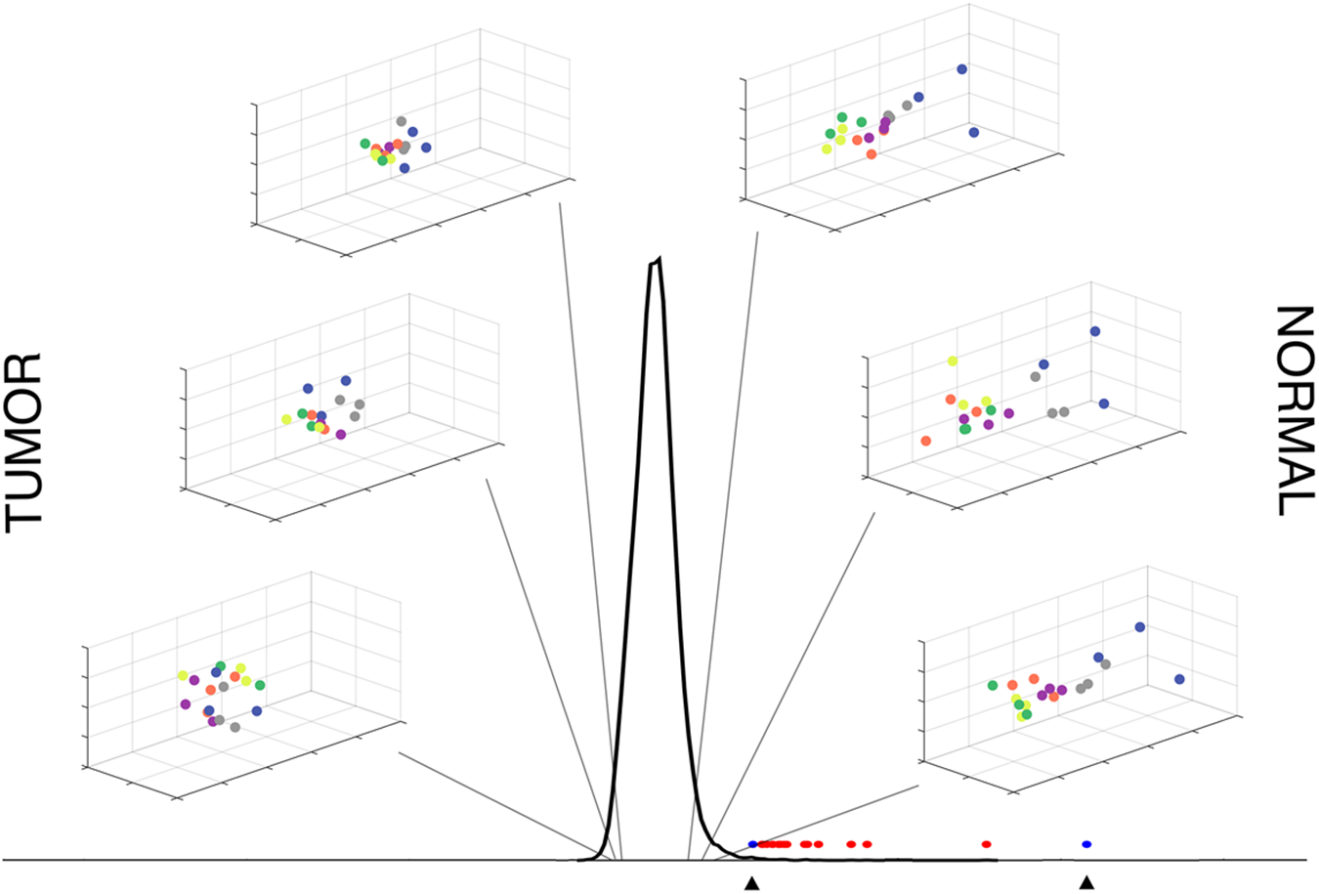
Molecular signature mutual information distinguishes cell groups. The MI(A,E)/MI(E,B) distribution in the center is for all molecular signatures. The lower ratios to the left of the distribution represent molecular signatures where the redirected state (E) is closer to the tumor state (B); The higher ratios to the right of the distribution represent molecular signatures where the redirected state (E) is closer to the normal state (A). Sample molecular signatures in the 3D insets show cell group replicate differences at percentiles 1, 2, 5 (left column, bottom to top) and 95, 98, 99 (right column, top to bottom) of the MI ratio distribution. Molecular signature markers: red markers at the base of the distribution indicate the position of the significant molecular signatures along the distribution; blue markers and arrows indicate the position of the most adversely affected molecular signature after the addition of the extended genes (left marker, PID_CERAMIDE_PATHWAY) and the molecular signature with the most improved ratio after extension (right marker, REACTOME_SOS_MEDIATED_SIGNALLING).

We visualized representative molecular signatures with CMDS at either extreme along the MI(A,E)/MI(E,B) distribution (Fig 4). Molecular signatures with the lowest and highest ratios correspond to signatures that associate with tumor versus normal states.

## Discussion

In this report, we describe the transcriptome profiling of *in vitro* samples modeling normal, tumor, and redirected cellular states. This analysis yielded 32,399 differentially expressed transcripts between the six cell states A-F (adjusted p < 0.0001; S1 Table). Each of these *single gene* differential expression patterns is interesting and a potential biomarker, especially those transcripts which were significantly different between redirected and tumor states but not between normal and redirected states. However, we found the large number of relevant transcripts to be unwieldy, so we chose to approach the biomarker discovery from a *polygenic* perspective using curated molecular signature gene sets that were significantly different in terms of mutual information between redirected and tumor states.

On the path to mutual information-based biomarker discovery, we observed that the global gene expression distributions were quite interesting. First, we noticed that the normal/redirected states demonstrated a bimodal distribution suggesting two distinct expression intensity gene populations (Fig 3A). The bimodal distribution dissolves as the expression state becomes more tumor-like and highly expressed genes shift to a lower expression state. It is possible that this shift is due to an increase in silenced tumor suppressor genes. Second, by determining the distance between global expression distributions with mutual information, we were able to sort the replicate groups into an alignment that satisfies intuition, where normal (A) and redirected (E) states are spatially close to each other but distant from tumor (B) states with some fluctuation in the intermediates, D, C, and F (Fig 3A). Since the global expression patterns were logical, we were confident that we could detect the specific gene sets underlying the states.

An excellent source of interdependent gene sets exists in the Broad Institute’s Molecular Signatures (MS) Database. Using the MI(A,E)/MI(E,B) ratio as a metric, we were able to filter over 13,000 MS down to 20—a manageable gene number that still maintains a significant level of the polygenic complexity required for tumorigenesis and presumably cancer cell redirection (Table 1). The sum of genes in these pathways is a collective biomarker set for cancer cell redirection. While the total unique gene count is high (906 genes), this number can be reduced to core genes that are not differentially expressed in normal (A) and redirected (E) states but are differentially expressed between normal and tumor (B) states and redirected and tumor states (107 genes, adjusted p < 0.0001). These core genes can be extended by the addition of 13 more, identified by on the basis of gene interactions, giving a total 120 of these core biomarkers. The full list of these is available in S6 Table. Thus, we have significantly narrowed the search for genes potentially involved in cancer cell redirection.

Most of the collective function of genes in the 20 signatures make sense in terms of tumor involvement and are candidate effectors for returning a tumor to a normal state. Eight signatures are clearly involved in cancer. One signature is involved in general tumorigenesis (INGA_TP53_TARGETS; 15 genes) and seven others in specific tumor subtypes: GOTZMANN_EPITHELIAL_TO_MESENCHYMAL_TRANSITION_UP (66 genes; liver cancer), MOSERLE_IFNA_RESPONSE (22 genes; ovarian cancer), CROMER_METASTASIS_DN (8 genes; head and neck cancer), BOGNI_TREATMENT_RELATED_MYELOID_LEUKEMIA_DN (28 genes; leukemia), LANDIS_BREAST_CANCER_PROGRESSION_UP (42 genes; breast cancer), HUANG_DASATINIB_RESISTANCE_UP (73 genes; breast cancer), and NIKOLSKY_BREAST_CANCER_21Q22_AMPLICON (13 genes; breast cancer). The latter three signatures are of high interest given the breast cancer model system we are investigating. While a full discussion of this large number of genes is too vast, we discuss two of the significant signatures in detail below.

**Fig 5:**
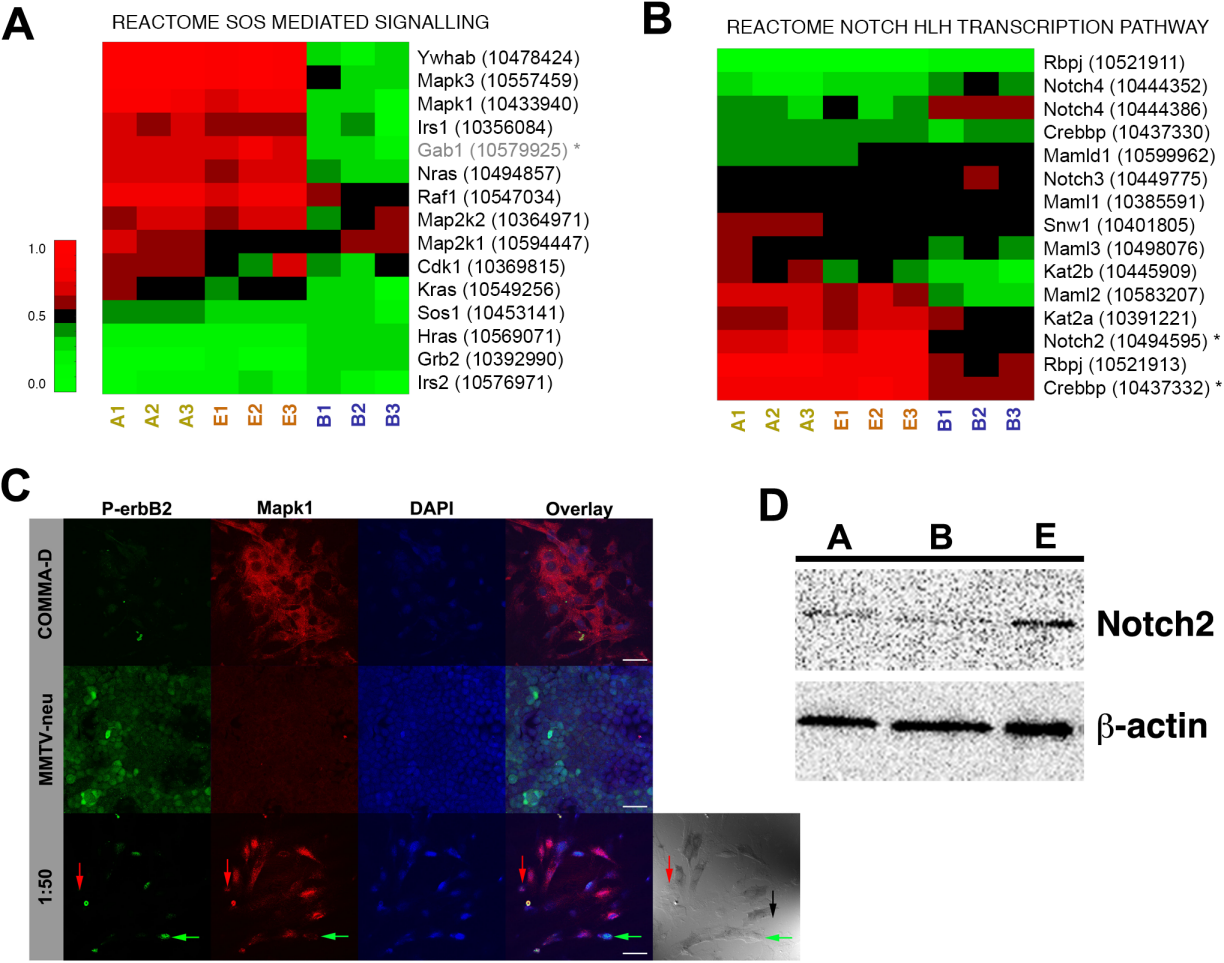
Representative cancer redirection molecular signatures. Markers indicate genes with at least one transcript identified as significantly differentially expressed. Gene names in gray are those added by interaction with the original molecular signatures members. Heatmaps for all groups are available in Supplemental Dataset 1. (A) Molecular signature of the SOS mediated signaling pathway. (B) Molecular signature of the Notch HLH pathway. (C) Immunoflourescent staining of cells for P-erbB2 (green) and Mapk1 (red). Nuclei counterstained with DAPI. Scale bars = 40 ***μ***m. Lower right image is DIC image with Xgal stained lacZ^+^ cells evident. (D) Western blots probed for Notch2. ***β***-actin used as loading control. A-Normal cell lysates, B-Cancer cell lysates, E-Lysates from 1:50 cultures.

The SOS mediated signaling molecular signature (REACTOME_SOS_MEDIATED_SIGNALLING; Table 1) contains a group of genes implicated in the widely investigated Ras-Mek-Erk pathway, a MAPK (mitogen-activated protein kinase) mediated pathway understood to play a role in cell proliferation and survival. Constitutional activation of this pathway, because of mutation, is a feature of many cancers [24–25]. Stimulation by a growth factor of a receptor tyrosine kinase like EGFR or erbB2 enables the subsequent binding of Grb2 and Sos1 to the receptor, beginning a phosphorylation cascade. The finding that the well-characterized Sos-mediated Ras-Mek-Erk pathway, frequently altered in cancer cells, exhibits the most extreme MI ratio is a reasonable result. Many genes showed expression levels that were depressed in tumor cells as compared to normal and redirected samples (Fig 5A). Mapk1 expression was found elevated in Group A and in Group E compared to Group B (Fig 5A). When cultures were examined for Mapk1 we found high Mapk1 expression in Group A normal cells, very little Mapk1 expression in Group B cancer cells, and differential expression in 1:50 co-cultures (Fig 5C). The normal cells continue to express Mapk1 in the 1:50 co-cultures. Cancer cells expressed very little Mapk1 in the 1:50 co-cultures (Fig 5C green arrows). Redirected cancer cells expressed Mapk1 (Fig 5C red arrows) thus confirming the genetic data.

The molecular signature with the third highest MI ratio is the Notch-HLH pathway (REACTOME_NOTCH_HLH_TRANSCRIPTION_PATHWAY; Table 1). The Notch family regulates normal development and when expression patterns are altered developmental disorders, tumor initiation, and cancer progression result. In the normal mouse mammary gland, high levels of Notch3 and Notch4 are found in the terminal end buds (TEBs) that are present during ductal morphogenesis [26]. TEBs contain a subpopulation of duct-limited progenitor cells that also give rise to myoepithelial cells. Notch3 also serves as a marker of luminal progenitor cells [27]. Activation of Notch1 results in an expansion of mammary luminal progenitor cells [28]. Notch1 and Notch2 regulate asymmetric division of mammary progenitor cells during pubertal development [29]. Inhibition of Notch1 reduces cancer stem cell function, reduces expression of Notch target genes *HES1*, *HES5* and *HEY-L*, and reduces the population of CD44^Hi^CD24^low^, a population identified as cancer stem cells [30–32]. *Notch1* was not one of the genes identified during analysis (S1 Table). The shift in Notch expression patterns during redirection is not unexpected as the cells are converting from a tumorigenic phenotype to a normal developmental phenotype (Fig 5B). Protein analysis revealed Notch2 expressed by Group A and Group E with lower expression found in Group B (Fig 5D). This result is similar to the genetic expression data (Fig 5B).

Not only are mouse tumor-derived cells susceptible to redirection [8, 11] but human breast cancer and embryonic testicular carcinoma cells have been redirected [9–10]. Both human female and male derived cancer cells differentiated into functional breast epithelial cells in response to signals from the normal mouse mammary microenvironment [8–11]. This suggests that human cancers are reactive to controlling intercellular signals present in developing mammalian tissues. The signaling pathways identified here may also be involved in potential human cancer cell redirection and differentiation.

One limitation of the results outlined within is the presumption that the different behavior between the normal cells, the cancer cells, and the redirected cells is due to the expression of specific genes and RNAs. Multiple studies have demonstrated that RNA expression does not definitively correlate to protein expression. The processes of mRNA decay, translation, and protein degradation are important factors in addition to mRNA transcription in regards to steady-state protein expression [33–36].

A second limitation of the data presented here is that it is derived from a single cancer cell line. The MMTV-neu mouse model is an accepted model of human HER2^+^ breast cancer [37]. There are numerous subsets of human breast cancer; the six main groups are 1) Luminal A, 2) Luminal B, 3) HER2 enriched, and 4) claudin low, 5) normal-like, and 6) basal-like [13]. Future studies will examine the profiles of redirected human breast cancer cells in vitro and in vivo. The human basal-like TNBC cell line MDA-MB-231 has been redirected in vivo so the models we use are not species limited [10].

**Fig 6:**
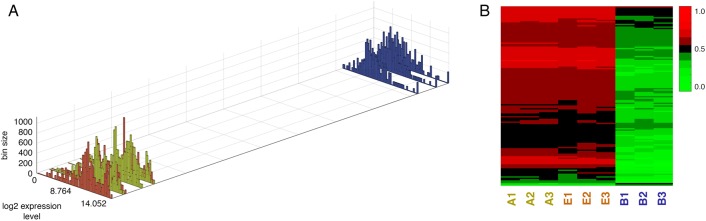
Core cancer redirection biomarker set. **A**: Expression level distributions 120 core genes visualized in 1D using CMDS; **B**: Heatmap of significantly differentially expressed transcripts of the 120 core genes.

In conclusion, to the best of our knowledge, we provide the first transcriptome profiling of an *in vitro* model system of cancer cell redirection. Through MI-based gene expression distribution sorting, we observe dramatic differences in global gene expression patterns between normal and tumor states as well as “movement” of intermediate states including cancer cell redirection towards the appropriate state (Fig 6). The twenty core molecular signatures we discovered using the MI-ratio approach are exciting candidate biomarker gene sets that could be controlling cancer cell redirection (Table 1). We are currently porting our system to human and *in vivo* systems to further delineate the causal genes that could hold the key to the genetic mechanism of cancer cell redirection.

## Supporting information

S1 Fig Validation of qRT-PCR. Real-time PCR validation of 3 target genes, ErbB2, AREG and THBS1. Relative expression based on expression of GAPDH.
(TIF)

S1 Table Complete analyses. Differential gene expression analysis between all combinations of cell groups.
(ZIP)

S2 Table List of primers. Primers used for gene analysis by quantitative real-time PCR.
(PDF)

S3 Table Extended genes associated with each of the significant molecular signatures and the basis of their interaction. (XLSX)

S4 Table All molecular signatures evaluated, ordered by decreasing MI(A,E)/MI(E,B) with member genes and probe set IDs. (ZIP)

S5 Table Results of extension of significant molecular signatures on MI(A,E)/MI(E,B) at several levels of stringency. (XLSX)

S6 Table Biomarker set. Genes added by interaction are indicated as well as those identified as *core* biomarkers are indicated.
(XLSX)
