# Supplementary figures and images for: Cancer cell redirection biomarker discovery using a mutual information approach
Source: PLoS One. 2017 Jun 8;12(6):e0179265. doi: 10.1371/journal.pone.0179265 (PMC5464651; doi:10.1371/journal.pone.0179265)

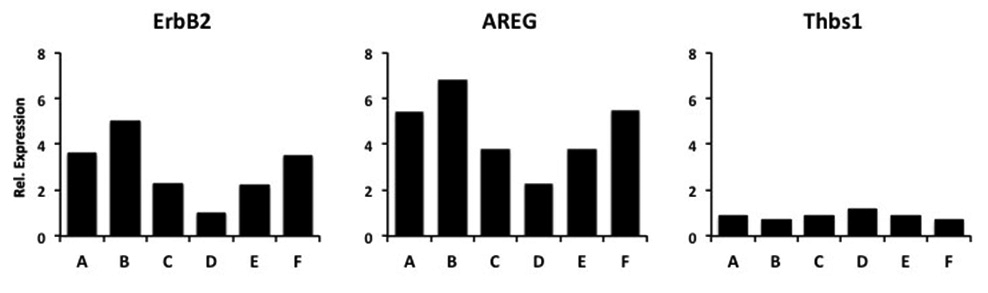

Supplement: S1 Fig — Real-time PCR validation of 3 target genes, ErbB2, AREG and THBS1. Relative expression based on expression of GAPDH. (TIF) [file pone.0179265.s001.tif]
