# Supplementary material for: Cancer cell redirection biomarker discovery using a mutual information approach
Source: PLoS One. 2017 Jun 8;12(6):e0179265. doi: 10.1371/journal.pone.0179265 (PMC5464651; doi:10.1371/journal.pone.0179265)
Supplement: S2 Table — Primers used for gene analysis by quantitative real-time PCR. (PDF) [file pone.0179265.s003.pdf]

Supplemental Table 6. Primers used for gene analysis by quantitative Real-Time PCR.

Gene	Sequence
AREG	1: 5’-CCTCCTTCTTTCTTCTGTTTCTCC-3’<br>2: 5’-GTCACTATCTTTGTCTCTGCCA-3’
ERBB2	1: 5’-ACAGACCAGAGTGCAGGAT-3’<br>2: 5’-CCTCATCACCTACAACACAGAC-3’
THBS1	1: 5’-ACCACGTTGCTGAATTCCAT-3’<br>2: 5’-AGTTCCTGATGGTGAATGCTG-3’
GAPDH	1: 5’-TGTAGTTGAGGTCAATGAAGGG-3’<br>2: 5’-ACATCGCTCAGACACCATG-3’
